# The mediating role of technology anxiety in the impact of loneliness on technology behavioral intention among community-dwelling older adults

**DOI:** 10.1371/journal.pone.0321144

**Published:** 2025-05-29

**Authors:** Chih-Yu Tsai, Ya-Ling Tzeng, Yu-Kuei Teng, Ting-Shan Chang

**Affiliations:** 1 School of Public Health, College of Public Health, China Medical University, Taichung, Taiwan; 2 School of Nursing, College of Health Care, China Medical University, Taichung, Taiwan; 3 Nursing Department, China Medical University Hospital, Taichung, Taiwan We greatly appreciate your understanding and assistance; Central China Normal University, CHINA

## Abstract

In this modern digital era, older adults encounter considerable challenges in technology adoption, primarily due to varying degrees of technology anxiety and its implications on their behavioral intentions. We conducted a cross-sectional survey with 250 participants aged 60 and older in Taiwan to investigate the mediating role of technology anxiety in the dynamic between loneliness and technology usage intentions. The results reveal that participants exhibited mild levels of loneliness (mean score: 1.53), moderate technology anxiety (mean score: 19.29), and moderate to high intentions toward technology use (mean score: 10.96). The study identifies a significant total effect of loneliness on technology behavioral intention (c = -0.508, p < 0.001). Specifically, loneliness was found to significantly influence technology anxiety (a = 1.158, p < 0.001), which in turn significantly impacted behavioral intention, even after adjusting for loneliness (b = -0.161, p < 0.001). Technology anxiety partially mediated the relationship between loneliness and behavioral intention, exacerbating its negative impact (c’ = -0.322, p < 0.001). These findings highlight the critical need for targeted interventions and policy measures to address and mitigate the barriers to technology adoption faced by older adults.

## 1. Introduction

In today’s digital age, technology plays an important role in older adults’ lives, influencing various aspects of their health and well-being. As the global population ages, ensuring the quality of life and social connectivity among older adults becomes increasingly vital. Technology serves as a tool for social interaction, entertainment, and information dissemination, bringing convenience but also a series of challenges. Unlike younger generations, older adults often encounter challenges in adopting technology due to factors such as limited technological literacy, physical changes related to aging, or unfamiliarity with new technologies. This technology anxiety (TA), driven by uncertainty or distrust in technological products or applications, can hinder their acceptance and utilization of technology, consequently impacting their behavioral intentions.

Instant messaging (IM) apps are among the most widely used communication software in Taiwan, particularly among individuals aged 60 to 69 (38.53%) and those aged 70 and above (12.78%) [1]. IM apps facilitate real-time and diverse interaction through text, voice, and video calls, providing a convenient platform for older adults to connect with family, friends, and community members [2]. Notably, during the pandemic, IM apps served as an alternative for older adults to engage in community activities, fostering a sense of participation. Therefore, it is critical to identify potentially modifiable factors associated with behavioral intentions in using IM apps when developing interventions to increase technology engagement among older adults.

The Technology Acceptance Model (TAM) offers a robust theoretical foundation for examining the formation of behavioral intentions. According to TAM, behavioral intention refers to an individual’s willingness to use a technology based on their subjective beliefs, serving as a key predictor of actual technology adoption and usage [3,4]. Technology acceptance is thus commonly conceptualized as the behavioral intention to adopt or utilize technology [5].

While TAM outlines various factors that influence behavioral intention, this study focuses on loneliness and technology anxiety as the primary determinants within the context of older adults, given that these are psychological and emotional factors commonly experienced in this population. By incorporating these dimensions into the theoretical framework, the study aims to advance understanding of the factors shaping older adults’ behavioral intentions toward technology use. Specifically, it highlights the mediating role of technology anxiety in the relationship between loneliness and behavioral intention, thereby addressing a critical gap in the existing literature.

Loneliness is a critical factor in the study of older adults’ technology use, directly impacting their quality of life and mental health [2,6–9]. As physical activity declines and chronic diseases rise with age, maintaining social connections becomes more challenging for older individuals. Factors such as retirement, the loss of spouses and friends, and changes in social structures contribute to increased loneliness and social isolation, negatively affecting overall health [7,9,10]. Research reveals that social interactions play a key role in enhancing the acceptance of technology among older adults [11]. Also, technology use can improve social connections and reduce loneliness in older adults [12]. While literature suggests that internet-based social activities can support and improve the social connections of older adults, the impact of loneliness on technology use outcomes remains inconsistent [7,9,13].

In the context of technology acceptance, technology anxiety emerges as a crucial determinant of behavioral intention, according to social cognitive theory [14]. Previous research indicates that technology anxiety is a significant predictor of technology use [15,16]. The research indicates that technology anxiety significantly reduces the intention to use smart devices for elderly individuals [17].It can influence an individual’s acceptance and use of information technology [15,18,19], particularly in studies related to older adults’ health information technology [18,20,21]. Research shows that “loneliness” and “technology anxiety” have an impact on “behavioral intentions”,respectively. Despite the accumulation of research on technology anxiety, loneliness, and behavioral intention among older adults [7,22–25], there is a gap in the literature that integrates their combined effects. This deficiency may hinder the development of effective improvement measures in the future. Therefore, by focusing on the comprehensive impact of technology anxiety, loneliness, and behavioral intention, this study aims to determine their influence on older adults’ use of IM apps. We hypothesize that (1) loneliness is positively correlated with technology anxiety, (2) loneliness is negatively correlated with behavioral intention, (3) technology anxiety is negatively correlated with behavioral intention, and (4) technology anxiety mediates the relationship between loneliness and behavioral intention.

The study seeks to understand the complex interplay affecting technology use among older adults by exploring these dynamics. The insights gained are expected to guide the development of targeted interventions and policies to effectively support the technology engagement of community-dwelling older adults.

## 2. Materials and methods

### 2.1. Study design

A cross-sectional survey was conducted from March 1 to May 31, 2023.

### 2.2. Setting and participants

The research participants consisted of 250 community-dwelling older adults, aged 60 years and above, from both urban and rural areas in central Taiwan. Convenience sampling was utilized for recruitment. Inclusion criteria required individuals to have previous or current experience using instant messaging applications. Exclusion criteria ruled out participants who were unable to communicate effectively, were illiterate, or were incapable of operating electronic devices.

This study used a paper-based version of the questionnaire, distributed by trained researchers at community centers and local events to ensure accessibility. Assistance was provided to participants who required help filling out the questionnaire. A pilot study was conducted to ensure that elderly participants could understand the questions as intended before the actual data collection. We explained the purpose and procedures of the study to the participants and obtained their consent prior to data collection. Participants were thanked for their involvement with small tokens of appreciation. The entire process was carried out with full anonymity and confidentiality to protect participants’ privacy.

### 2.3. Ethical considerations

The study was approved by the Research Ethics Committee of China Medical University (CMUH111-REC3-229), and written informed consent was obtained from all participants.

### 2.4. Data collection tools

Data were collected through a questionnaire survey, which included demographic variables, the 6-item De Jong Gierveld Loneliness Scale, the technology anxiety Scale, and the Behavioral Intention Scale.

### 2.5. 6-item De Jong Gierveld loneliness scale

The 6-item De Jong Gierveld Loneliness Scale is a widely used instrument recognized for its good reliability and validity across various populations, demonstrating its solid psychometric properties. For our research, we adopted the Chinese version translated by Leung et al. (2008) to assess loneliness among older adults. The scale consists of six items, with the total loneliness score ranging from 0 to 6. The higher the score on the scale, the higher the level of loneliness.

### 2.6. Technology anxiety scale

The technology anxiety scale used in this study was adapted from Chang, Chih-Ling (2016), referencing Raub (1981) and Agarwal and Karahanna’s (2000). Six items were retained from the original scale after factors with factor loading λ < 0.5 were eliminated. The scale employs a Likertscale with five response options: “Strongly Disagree,” “Disagree,” “Neutral,” “Agree,” and “Strongly Agree.” Each item is scored from 1 (Strongly Disagree) to 5 (Strongly Agree), with total scores ranging from 6 to 30. Higher agreement indicates higher levels of anxiety towards the specific item. The Cronbach’s α for this scale in the current study was 0.94.

### 2.7. Behavioral intention scale

Behavioral intention scale was used a part of the original TAM scale that was developed by Davis (1985) and further revised by Sung, Hsai-Mei (2011). The scale employs a Likert scale with five response options: “Strongly Disagree,” “Disagree,” “Neutral,” “Agree,” and “Strongly Agree.” Each item is scored from 1 (Strongly Disagree) to 5 (Strongly Agree). The behavioral intention scale consists of three items, with the total behavioral intention ranging from 3 to 15. Higher scores indicate higher agreement with the importance of the respective factors. In this study, the Cronbach’s α was 0.89 for behavioral intention, with all questionnaire reliability α coefficients exceeding 0.7.

### 2.8. Statistical analysis

Data were analyzed using SPSS 25.0 software (IBM Corporation, Armonk, NY). Categorical data were summarized as frequencies and percentages, while continuous data were presented as means with standard deviations (SD). Pearson’s correlation was applied to examine the relationships among loneliness, technology anxiety, and behavioral intention. Regression analysis was conducted using the SPSS PROCESS macro (version 4). All findings were evaluated at a significance level of p < 0.05.

**Fig 1:**
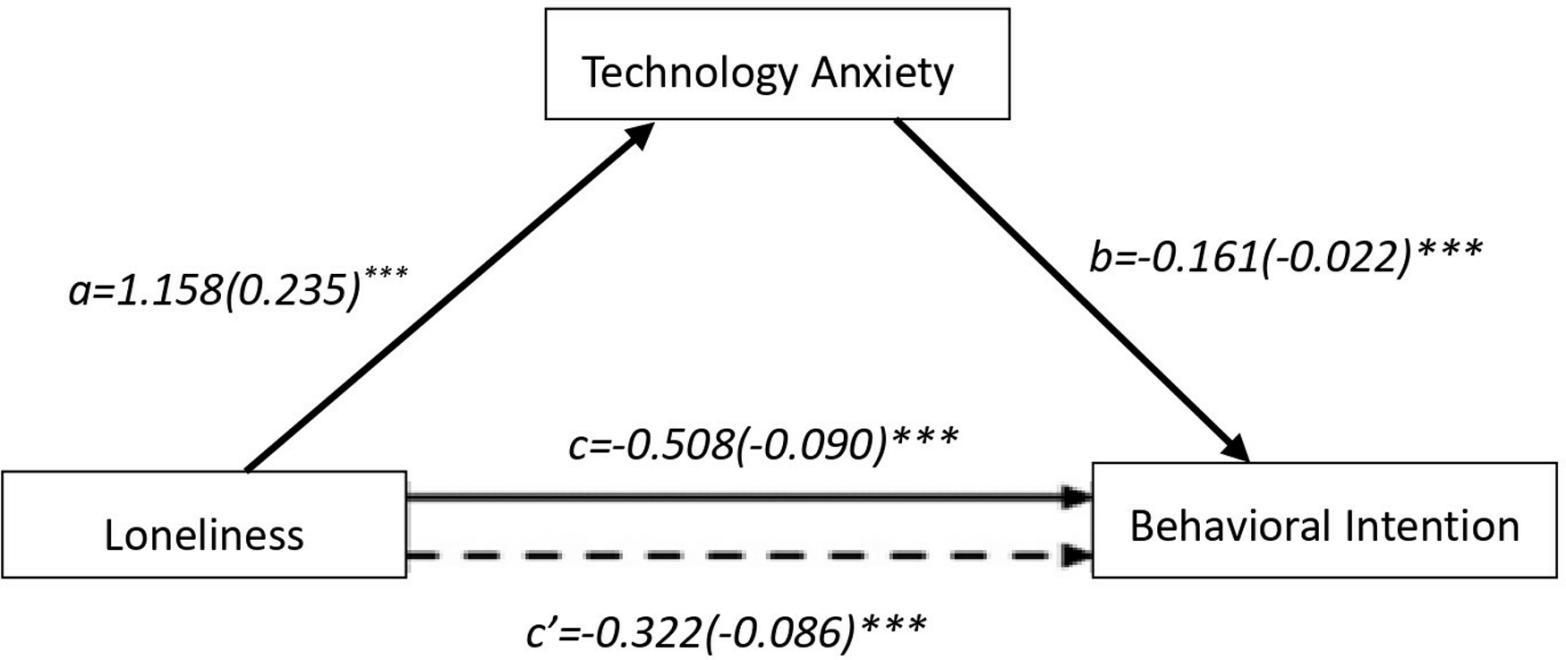
Mediation effects of technology anxiety on the relationship between loneliness and behavioral intention. The model shows the unstandardized path coefficients and the standard error values for each.

The mediation analysis framework assessed the effect of the independent variable on the dependent variable, considering the role of the mediator. In this study, loneliness was identified as the independent variable, technology anxiety as the mediator, and behavioral intention as the dependent variable (Fig 1).

Path a refers to the impact of loneliness (independent variable) on technology anxiety (mediator variable), while path b denotes the effect of technology anxiety on behavioral intentions (dependent variable). Path c (total effect) encompasses both the direct impact of loneliness on behavioral intentions (path c’) and the indirect impact of loneliness on behavioral intentions through technology anxiety (path a+b). To estimate the direct, indirect, and total effects, we employed5,000 bootstrap samples for a 95% bias-corrected bootstrap confidence interval (CI).

## 3. Results

### 3.1. Participants’ characteristics

The demographic characteristics of the study participants are systematically summarized in Table 1. The participants comprised a total of 250 individuals, with a mean age of 70.50 years (SD = 7.30 years). Gender distribution within the cohort was predominantly female, constituting 139 participants (55.60%), while male participants accounted for 111 (44.40%). Regarding educational attainment, the most common level was high school, represented by 64 individuals (25.70%). In terms of monthly income, a significant proportion of participants reported earnings ranging from 20,000 to 29,999 new Taiwan dollar (NTD), with 63 individuals (25.40%) falling into this category. Residential areas were fairly evenly distributed between urban and rural locales, with urban dwellers making up 126 participants (50.40%) and rural residents accounting for 124(49.60%).

**Table 1:** Participants’ characteristics (n = 250).

characteristics	Group	Frequency (N)	Percentage (%)
Age(years)	60-64	63	25.20
	65-69	59	23.60
	70-74	51	20.40
	75-79	38	15.20
	≧80	39	15.60
Gender	Male	111	44.40
	Female	139	55.60
Education	≦Elementary school	53	21.29
	Junior high school	60	24.10
	high school	64	25.70
	specialist	40	16.06
	≧University	32	12.85
Income (NTD, monthly)	≦19999	56	22.58
	20000-29999	63	25.40
	30000-39999	54	21.77
	40000-49999	25	10.08
	50000-59999	20	8.06
	≧60000	26	10.48
	Other	4	1.61
Living area	City	126	50.40
	Country	124	49.60

### 3.2 Correlations of behavioral intention, technology anxiety, and loneliness

As illustrated in Table 2, the study investigated the correlations among loneliness, technology anxiety, and behavioral intention. The mean scores for loneliness, technology anxiety, and behavioral intention were 1.53 (SD = 1.50), 19.29 (SD = 5.82), and 10.96 (SD = 2.26), respectively. Pearson’s correlation analysis was used to examine the interrelations among these variables. The results revealed a significant positive correlation between technology anxiety and loneliness (r = 0.30, p < 0.01), indicating that higher levels of technology anxiety are associated with higher levels of loneliness. Conversely, loneliness was found to be negatively correlated with behavioral intention (r = -0.34, p < 0.01), suggesting that individuals with higher levels of loneliness are likely to exhibit lower levels of behavioral intention. Additionally, technology anxiety was significantly negatively correlated with behavioral intention (r = -0.48, p < 0.01), indicating that increased technology anxiety is associated with lower behavioral intention.

**Table 2:** Correlations between the main study variables.

Variables	Mean	SD	Loneliness	Technology anxiety	Behavioral intention
Loneliness	1.53	1.50	1		
Technology anxiety	19.29	5.82	0.30^**^	1	
Behavioral intention	10.96	2.26	-0.34^**^	-0.48^**^	1

** The correlation is significant at the 0.01 level (two-tailed).

### 3.3 Mediation analysis of loneliness, technology anxiety, and behavioral intention

The model four of PROCESS macro was adopted to test the mediation effect. Fig 1 illustrated the mediation model, along with unstandardized path coefficients and standard error values for each. As presented in Table 3, the results indicated that the total effect of loneliness on behavioral intention was significant (c= -0.508, SE= -0.090, 95% CI [-0.686, -0.331], p<0.001). The effects from loneliness to technology anxiety (a = 1.158, p < 0.001) and technology anxiety to behavioral intention after adjustment for loneliness (b = -0.161, p<0.001) was also significant. The effect of loneliness on behavioral intention through technology anxiety was the product of these two paths (ab = -0.186.95% bias-corrected boot strap CI [-0.2997, -0.0948]). The ab pathway indicates that the indirect effect of loneliness on behavioral intention through technology anxiety is negative (-0.186), which means that loneliness increases the level of technology anxiety (positive effect, a > 0), while technology anxiety decreases behavioral intention (negative effect, b < 0). The bootstrapping results show 95% confidence interval between -0.2997 and -0.0948 for the indirect effect that does not include zero, confirming that the indirect effect is statistically significant. After adjustment for technology anxiety. The effects of loneliness on behavioral intention were also significant (c’= -0.322, p<0.001), indicating that loneliness hare an indirect negative effect on behavioral intention by reducing technology anxiety for community dwelling older adults’ total mediation.

**Table 3:** mediation analysis (N = 250).

variables	B	SE	t	*p*	LL95%CI	UL95%CI
Loneliness→Technology anxiety	1.158	0.235	4.917	<.001	0.694	1.622
Technology anxiety→Behavioral intention	-0.161	0.022	-7.274	<.001	-0.204	-0.117
Loneliness→Behavioral intention	-0.508	0.090	-5.637	<.001	-0.686	-0.331
Loneliness→Technology anxiety→Behavioral intention	-0.322	0.086	-3.749	<.001	-0.491	-0.153

## 4. Discussion

This study found that loneliness significantly reduces older adults’ behavioral intention to use technology, both directly and indirectly, through increased technology anxiety. The results highlight the psychological barriers that affect technology adoption among older adults, providing new insights into the factors influencing their behavioral intention. The discussion is structured into several key points: Loneliness as a Key Psychological Factor Affecting Technology Use, Technology Anxiety as a Modifiable Barrier, Behavioral Intention and Technology Design for Older Adults, Revisiting the TAM in the Context of Older Adults, and Toward an Inclusive Digital Future for Older Adults.

### Loneliness as a key psychological factor affecting technology use.

This study explored how technology anxiety mediates the relationship between loneliness and behavioral intention. Rather than being a mere emotional state, loneliness contributes to increased technology anxiety, which in turn diminishes older adults’ willingness to use digital tools. These findings suggest that loneliness is not merely an emotional state but also a psychological driver that influences technology acceptance behavior, especially as technology increasingly serves as a tool for social interaction.

The study found a positive correlation between loneliness and technology anxiety, supporting the hypothesis (H1) that loneliness may lead to fear and discomfort with technology. Additionally, loneliness was negatively correlated with older adults’ intention to use instant messaging applications, aligning with hypothesis (H2), which suggests that loneliness may reduce willingness to adopt technology by amplifying negative emotions or decreasing perceptions of technological efficacy. These results not only support prior research on the negative impact of loneliness on social behavior but also emphasize its profound influence on digital technology acceptance, indicating that loneliness should be incorporated into theoretical frameworks related to technology adoption.

### Technology anxiety appears to be a modifiable barrier to technology adoption among older adults

The negative correlation between technology anxiety and behavioral intention also supports hypothesis (H3), indicating that technology anxiety reduces older adults’ willingness to use technology and may become a barrier to adoption. Within the framework of the Technology Acceptance Model (TAM), anxiety about technology might lead older adults to perceive technology as difficult to use, thereby reducing their willingness to adopt it [15,17–21]. Finally, technology anxiety significantly mediated the relationship between loneliness and behavioral intention, showing that loneliness and behavioral intention are indirectly negatively correlated through the influence of technology anxiety, supporting hypothesis (H4). This finding underscores that technology anxiety is not only a barrier to behavioral intention but also a critical intermediary through which loneliness impacts the technology acceptance process.

Technology anxiety is a key factor associated with the behavioral intention of community-dwelling older adults, consistent with a previous study indicating a negative correlation between technology anxiety and behavioral intention among community-dwelling older adults [7,22–25]. Our results show that community-dwelling older adults with lower levels of technology anxiety exhibit higher behavioral intention to use instant messaging software. The degree of technology anxiety affects the experience of using technology; higher levels of technology anxiety result in fewer benefits derived from technology [24]. Furthermore, lower levels of technology anxiety imply more social contact and greater utilization of healthcare services, helping community-dwelling older adults access more health-related information and skills, thereby increasing their behavioral intention. This suggests that technology anxiety is a modifiable psychological factor that can be mitigated through appropriate interventions, such as training programs or social support, providing concrete directions for improving older adults’ technology acceptance.

### Older adults' behavioral intention to use instant messaging software averaged 3.65 on a 5-point scale, reflecting a moderate level of intention.

Specifically, the item “I would recommend using instant messaging software with family and friends” received the lowest score, while “Instant messaging software will be a part of my life in the future” received the highest score. These findings suggest varying degrees of acceptance and readiness to endorse the use of this technology among participants. This variation may reflect psychological barriers among older adults in adopting social technologies, such as distrust of new technology, concerns about privacy, and the complexity of system operations.

Older adults’ adoption and use of technology products may be influenced by various factors, including aging-related conditions, affordability issues, lack of digital literacy, operational difficulties with communication technologies, concerns about privacy and security, and challenges posed by unfriendly system interfaces [18,26]. Each of these factors may negatively impact behavioral intentions. To address these challenges, technology designers should consider older adults’ needs by improving interface usability and developing targeted educational programs to lower learning barriers and enhance technology usability.

Consistent with previous research, the use of technology products by older adults has been found to potentially reduce loneliness [7,9,13]. This aligns with observations that community-dwelling older adults with lower levels of loneliness tend to show greater willingness and ability to engage with instant messaging software [7]. However, there are also studies indicating that the differences in loneliness levels before and after adopting technology among older adults are not significant [27–29]. This disparity in findings could suggest that while technology has the potential to alleviate loneliness, the effectiveness may depend on various factors such as the subjectivity of loneliness, the individual’s initial level of loneliness, the type of technology used, and how it is integrated into their daily routines. Further research is needed to explore these variables and to determine under what conditions technology can be most beneficial in reducing loneliness among older adults.

### A deeper examination of the Technology Acceptance Model (TAM) in the context of older adultsoffers additional insight into the findings.

The Technology Acceptance Model (TAM) emphasizes two key constructs—perceived ease of use and perceived usefulness—as primary drivers of technology adoption [30]. The findings of this study demonstrate that psychological factors, particularly loneliness and technology anxiety, play pivotal roles in shaping these perceptions and behavioral intention, ultimately influencing older adults’ technology adoption. This challenges TAM’s focus on cognitive constructs and highlights the importance of emotional and social influences. Loneliness indirectly reduces behavioral intention by increasing technology anxiety, suggesting that psychological states may precede and influence the TAM constructs of perceived ease of use and perceived usefulness. For example, increased technology anxiety caused by loneliness may amplify fears and discomfort associated with technology, reducing perceived ease of use and subsequently lowering behavioral intention. Unlike younger populations, where perceived usefulness may dominate technology adoption decisions, older adults’ behavioral intentions appear to be more influenced by emotional barriers such as loneliness and anxiety.

However, TAM 2, as extended by Venkatesh and Davis (2000), incorporates additional influencing factors, such as subjective norm and image as key determinants of perceived usefulness [31]. This study indirectly supports these extensions. For older adults with heightened loneliness, a lack of social support may diminish the effects of subjective norm and image, contributing to a decrease in technology adoption intention. Furthermore, TAM 3, developed by Venkatesh and Bala (2008) as an extension of TAM 2, introduced constructs such as computer self-efficacy, perceptions of external control, anxiety, and playfulness [32]. The inclusion of technology anxiety and self-efficacy in TAM 3 aligns closely with the findings of this study. Technology anxiety significantly reduces older adults’ perceived ease of use, thereby diminishing their behavioral intention, which is consistent with the assumptions of TAM 3.

Although TAM 2 and TAM 3 enhance the explanatory power of technology adoption behavior, they remain largely focused on cognitive constructs such as perceived usefulness and ease of use. This study challenges this cognitive emphasis by highlighting the central role of emotional and psychological factors, such as loneliness and anxiety, in technology adoption. Specifically, among older adults, the influence of loneliness and technology anxiety on behavioral intention is substantial and may even surpass the impact of perceived usefulness and subjective norms. These findings suggest that extended versions of TAM should further incorporate psychological and emotional factors, particularly when applied to special populations like older adults.

Future expansions of the model could consider integrating psychological factors, accounting for contextual and cultural differences, and balancing cognitive and emotional constructs to provide a more comprehensive explanation of technology acceptance behavior among older adults.

### To support future directions in designing for aging populations, we must also consider how broader inclusion goals can be achieved

As the global population ages, older adults face unique challenges, including diminishing social networks, which make technology adoption increasingly crucial for maintaining connections and accessing resources. This study highlights the dual impact of loneliness and technology anxiety on behavioral intention, emphasizing the need for targeted interventions to improve older adults’ acceptance of technology. Developing technology training programs tailored to the specific needs of older adults can help build confidence and reduce fears associated with digital tools. Moreover, fostering social support from family, friends, and communities can alleviate technology anxiety, enabling older adults to overcome barriers and engage more effectively with technology. Simplifying system designs to address usability and privacy concerns can further enhance trust and encourage adoption.

A research support more social support from family and friends around their communication technology use, resulting in decreased technophobia and social isolation after intervention [33]. Future research could focus on the cultural contexts of older adults, individual variations in loneliness, and the impact of different types of technology on these outcomes. Understanding the effectiveness of various intervention strategies across diverse populations will provide valuable insights for developing inclusive policies and designing technologies that cater to the specific needs of older adults. These strategies aim to bridge the digital divide, ensuring that older adults benefit from technological advancements while improving their quality of life and social connections. By addressing both psychological and practical challenges, a more inclusive digital environment can be developed to better support older adults in navigating and benefiting from an increasingly technology-driven society.

## 5. Strengths and limitations

The study benefits from several strengths that enhance the validity and relevance of its findings. Importantly, it includes a diverse participants of older adults from both urban and rural areas in central Taiwan, ensuring a range of environmental contexts are represented. The sampling strategy was carefully designed to maintain a balance in gender and age distribution, which adds depth to the analysis of technology usage across different demographic groups. Moreover, by focusing on the relationship between loneliness, technology anxiety, and behavioral intentions specifically related to instant messaging software, the study fills a critical gap in the literature. This focus is particularly pertinent given the potential of this technology to facilitate increased social interaction and connectivity among older adults.

Despite these strengths, the study’s methodology entails certain limitations that must be acknowledged. The cross-sectional design, while useful for identifying associations between variables at a single point in time, does not allow for conclusions about causality or changes over time. Longitudinal research would be necessary to determine the directions of these relationships and to observe how they evolve. Furthermore, although the participants include both urban and rural residents, it does not encompass the older adult population across all counties in Taiwan, which may limit the generalizability of the findings to all older adults in Taiwan. Additionally, the reliance on self-reported data could introduce response biases, as participants might modify their responses based on social desirability or misremember past behaviors and feelings.

## 6. Conclusion

This study demonstrates that technology anxiety among community-dwelling older adults plays a partial mediating role in the relationship between loneliness and behavioral intention. These findings contribute to understanding the mechanisms by which loneliness and technology anxiety affect behavioral intention, and provide recommendations for precise intervention measures and appropriate policy formulation in the future. Interventions that effectively reduce loneliness and address technology anxiety may significantly enhance the willingness of older adults to engage with technology, ultimately fostering greater inclusivity and connectivity within this demographic.

## Supporting information

S1 Data Database PONE-D-24-27931. (SAV)

## References

[1] Center TNI. 2022 Taiwan Internet Report-Overall Internet Usage. Available from: https://twnic.tw/stat_n.php

[2] Fan Q. Utilizing ICT to prevent loneliness and social isolation of the elderly. A literature review. Cuad Trab Soc. 2016;29(2):185–200. doi: 10.5209/cuts.51771

[3] King WR, He J. A meta-analysis of the technology acceptance model. Information & Management. 2006;43(6):740–55. doi: 10.1016/j.im.2006.05.003

[4] Granić A, Marangunić N. Technology acceptance model in educational context: A systematic literature review. Brit J Educational Tech. 2019;50(5):2572–93. doi: 10.1111/bjet.12864

[5] Zhang S, Boot WR. Predicting Older Adults’ Continued Computer Use After Initial Adoption. Innov Aging. 2023;7(4):igad029. doi: 10.1093/geroni/igad029 37197443 PMC10184684

[6] Chiu C-J, Hu Y-H, Lin D-C, Chang F-Y, Chang C-S, Lai C-F. The attitudes, impact, and learning needs of older adults using apps on touchscreen mobile devices: Results from a pilot study. Computers in Human Behavior. 2016;63:189–97. doi: 10.1016/j.chb.2016.05.020

[7] Blažun H, Saranto K, Rissanen S. Impact of computer training courses on reduction of loneliness of older people in Finland and Slovenia. Computers in Human Behavior. 2012;28(4):1202–12. doi: 10.1016/j.chb.2012.02.004

[8] Casanova G, Zaccaria D, Rolandi E, Guaita A. The Effect of Information and Communication Technology and Social Networking Site Use on Older People’s Well-Being in Relation to Loneliness: Review of Experimental Studies. J Med Internet Res. 2021;23(3):e23588. doi: 10.2196/23588 33439127 PMC7961406

[9] Chen K, Chan AHS. A review of technology acceptance by older adults. Gerontechnology. 2011;10(1). doi: 10.4017/gt.2011.10.01.006.00

[10] Quinn K. Cognitive Effects of Social Media Use: A Case of Older Adults. Soc Media Soc. 2018;4(3):10.1177/2056305118787203. doi: 10.1177/2056305118787203 37041879 PMC10085578

[11] Lüders M, Gjevjon ER. Being old in an always-on culture: Older people’s perceptions and experiences of online communication. The Information Society. 2017;33(2):64–75. doi: 10.1080/01972243.2016.1271070

[12] Czaja SJ, Boot WR, Charness N, Rogers WA, Sharit J. Improving Social Support for Older Adults Through Technology: Findings From the PRISM Randomized Controlled Trial. Gerontologist. 2018;58(3):467–77. doi: 10.1093/geront/gnw249 28201730 PMC5946917

[13] Casanova G, Zaccaria D, Rolandi E, Guaita A. The Effect of Information and Communication Technology and Social Networking Site Use on Older People’s Well-Being in Relation to Loneliness: Review of Experimental Studies. J Med Internet Res. 2021;23(3):e23588. doi: 10.2196/23588 33439127 PMC7961406

[14] Yang K, Forney J. The moderating role of consumer technology anxiety in mobile shopping adoption: Differential effects of facilitating conditions and social influences. J Electron Commerce Res. 2013;14(4):334.

[15] Purnomo SH, Lee Y-H. E-learning adoption in the banking workplace in Indonesia. Information Development. 2012;29(2):138–53. doi: 10.1177/0266666912448258

[16] Czaja S, Charness N, Dijkstra K, Fisk A, Rogers W, Sharit J. CREATE common core battery of measures: Technical report No. CREATE-2006-01. Center for Research and Education on Aging and Technology Enhancement (CREATE), Atlanta, GA. 2006 10.1037/0882-7974.21.2.333 PMC1524856 16768579

[17] Jeng M-Y, Pai F-Y, Yeh T-M. Antecedents for Older Adults’ Intention to Use Smart Health Wearable Devices-Technology Anxiety as a Moderator. Behav Sci (Basel). 2022;12(4):114. doi: 10.3390/bs12040114 35447686 PMC9028451

[18] Mo Y, Deng L, Zhang L, Lang Q, Liao C, Wang N, et al. Work stress among Chinese nurses to support Wuhan in fighting against COVID-19 epidemic. J Nurs Manag. 2020;28(5):1002–9. doi: 10.1111/jonm.13014 32255222 PMC7262235

[19] Guner H, Acarturk C. The use and acceptance of ICT by senior citizens: a comparison of technology acceptance model (TAM) for elderly and young adults. Univ Access Inf Soc. 2018;19(2):311–30. doi: 10.1007/s10209-018-0642-4

[20] Ma Q, Chan AHS, Chen K. Personal and other factors affecting acceptance of smartphone technology by older Chinese adults. Appl Ergon. 2016;54:62–71. doi: 10.1016/j.apergo.2015.11.015 26851465

[21] Macedo IM. Predicting the acceptance and use of information and communication technology by older adults: An empirical examination of the revised UTAUT2. Computers in Human Behavior. 2017;75:935–48. doi: 10.1016/j.chb.2017.06.013

[22] Su SW. *The influence of technology anxiety and lifestyle on consumer use and experiences with mobile commerce*. Fo Guang University College of Humanities; 2004. doi: hdl.handle.net/11296/juy38r

[23] Chen K, Chan AHS. Gerontechnology acceptance by elderly Hong Kong Chinese: a senior technology acceptance model (STAM). Ergonomics. 2014;57(5):635–52. doi: 10.1080/00140139.2014.895855 24655221

[24] Meuter ML, Ostrom AL, Bitner MJ, Roundtree R. The influence of technology anxiety on consumer use and experiences with self-service technologies. Journal of Business Research. 2003;56(11):899–906. doi: 10.1016/s0148-2963(01)00276-4

[25] Fischer SH, David D, Crotty BH, Dierks M, Safran C. Acceptance and use of health information technology by community-dwelling elders. Int J Med Inform. 2014;83(9):624–35. doi: 10.1016/j.ijmedinf.2014.06.005 24996581 PMC4144164

[26] Lin YH, Lin SJ. Digital divides revisited: a process view of the acquisitions of information and communication technology (ICT) skills by the elderly. J Libr Inf Sci Res. 2009;3(2):75–102.

[27] Morton TA, Wilson N, Haslam C, Birney M, Kingston R, McCloskey L-G. Activating and Guiding the Engagement of Seniors With Online Social Networking: Experimental Findings From the AGES 2.0 Project. J Aging Health. 2018;30(1):27–51. doi: 10.1177/0898264316664440 27530332

[28] Ferguson L, Kürüm E, Rodriguez TM, Nguyen A, Lopes de Queiroz IF, Lee J, et al. Impact of community-based technology training with low-income older adults. Aging Ment Health. 2024;28(4):638–45. doi: 10.1080/13607863.2023.2256271 37702149

[29] Myhre JW, Mehl MR, Glisky EL. Cognitive Benefits of Online Social Networking for Healthy Older Adults. J Gerontol B Psychol Sci Soc Sci. 2017;72(5):752–60. doi: 10.1093/geronb/gbw025 26984523

[30] Davis FD. Perceived Usefulness, Perceived Ease of Use, and User Acceptance of Information Technology. MIS Quarterly. 1989;13(3):319. doi: 10.2307/249008

[31] Venkatesh V, Davis FD. A Theoretical Extension of the Technology Acceptance Model: Four Longitudinal Field Studies. Management Science. 2000;46(2):186–204. doi: 10.1287/mnsc.46.2.186.11926

[32] Venkatesh V, Bala H. Technology Acceptance Model 3 and a Research Agenda on Interventions. Decision Sciences. 2008;39(2):273–315. doi: 10.1111/j.1540-5915.2008.00192.x

[33] Fang Y, Chau AKC, Wong A, Fung HH, Woo J. Information and communicative technology use enhances psychological well-being of older adults: the roles of age, social connectedness, and frailty status. Aging Ment Health. 2018;22(11):1516–24. doi: 10.1080/13607863.2017.1358354 28777010

